# Breastfeeding knowledge, attitude and practice among school teachers in Abha female educational district, southwestern Saudi Arabia

**DOI:** 10.1186/1746-4358-7-10

**Published:** 2012-08-15

**Authors:** Ali Mohamed Al-Binali

**Affiliations:** 1 Department of Child Health, College of Medicine, King Khalid University, P.O. Box 641, 61421, Abha, Kingdom of Saudi Arabia

**Keywords:** Breastfeeding, Knowledge, Practice, Attitude, School teachers

## Abstract

**Background:**

Inadequate knowledge, or inappropriate practice, of breastfeeding may lead to undesirable consequences. The aim of this study was to assess breastfeeding knowledge, attitude and practice (KAP) among female teachers in the Abha Female Educational District and identify factors that may affect breastfeeding practice in the study population.

**Methods:**

A cross-sectional study using a self-administered questionnaire was conducted among school teachers in Abha Female Educational District during the months of April to June, 2011. Breastfeeding KAP of participants who had at least one child aged five years or younger at the time of the study were assessed using a self-administered questionnaire, based on their experience with the last child.

**Results:**

A total of 384 women made up of 246 (61.1%) primary-, 89 (23.2%) intermediate- and 49 (12.8%) high-school teachers participated in the study. One hundred and nineteen participants (31%) started breastfeeding their children within one hour of delivery, while exclusive breastfeeding for 6 months was reported only by 32 (8.3%) participants. Insufficient breast milk and work related problems were the main reasons given by 169 (44%) and 148 (38.5%) of participants, respectively, for stopping breastfeeding before two years. Only 33 participants (8.6%) had attended classes related to breastfeeding. However, 261 participants (68%) indicated the willingness to attend such classes, if available, in future pregnancies.

**Conclusions:**

This study revealed that breast milk insufficiency and adverse work related issues were the main reasons for a very low rate of exclusive breastfeeding among female school teachers in Abha female educational district, Saudi Arabia. A very low rate of attending classes addressing the breastfeeding issues during pregnancy, and an alarming finding of a high percentage of babies receiving readymade liquid formula while still in hospital, were also brought out by the present study. Such findings, if addressed comprehensively by health care providers and decision-makers, will lead to the improvement of breastfeeding practices in the study community.

## Background

Breastfeeding is an important public health strategy for improving infant and child morbidity and mortality, improving maternal morbidity, and helping to control health care costs. Breastfeeding is associated with a reduced risk of otitis media, gastroenteritis, respiratory illness, sudden infant death syndrome, necrotizing enterocolitis, obesity, and hypertension [1]. The World Health Organization (WHO) and United Nations Children’s Fund (UNICEF) recommend that every infant should be exclusively breastfed for the first six months of life, with breastfeeding continuing for up to two years of age or longer [2-4]. Exclusive breastfeeding is defined as feeding the infant only breast milk, with no supplemental liquids or solids except for liquid medicine and vitamin/mineral supplements [4].

Variables that may influence breastfeeding include race, maternal age, maternal employment, level of education of parents, socio-economic status, insufficient milk supply, infant health problems, maternal obesity, smoking, parity, method of delivery, maternal interest and other related factors [5,6].

A number of studies have addressed breastfeeding in different parts of Saudi Arabia in respect of relevant statistics, factors influencing breastfeeding and attitudes towards breastfeeding [7-12], but virtually no data have been reported on breastfeeding among Saudi female school teachers. Female school teachers compromise half the female workforce in Saudi Arabia – 250,854 teachers out of 505,340 female employees – and they exert a tremendous socializing influence on up-coming generations [13]. Dissemination of breastfeeding knowledge to this population should have a striking impact on child health in the country in the short-to-medium term, as well as in the more distant future, since teachers are the best suited to pass on correct attitudes to the mothers of tomorrow [6]. The present study was undertaken, therefore, to assess breastfeeding knowledge, attitude and practice (KAP) of female school teachers in Abha educational district, and to compare them against international standards, identify factors which mitigate breastfeeding KAP and make suggestions for minimizing obstacles to satisfactory breastfeeding.

## Methods

### Study setting and population

This was a postal survey conducted among female school teachers at Abha Female Educational District, Southwestern, Saudi Arabia between April and June 2011.

The target study population was female school teachers with the youngest child aged 5 years or less.

It was anticipated that less than 25% of nursing mothers in the study population breastfed exclusively. With absolute precision of 5% at 95% confidence interval, the minimum sample size required for the study was calculated to be 289 mothers [14]. The questionnaires were distributed to 400 women in order to achieve our target of at least 289 participants.

### Study instrument

In the absence of a validated standardized questionnaire, the screening tool used in the present study was prepared using Delphi technique. A group of experts (from the College of Medicine, King Khalid University) in the fields of Pediatrics, Child Nutrition, Community Medicine and Behavioral Sciences designed and produced the preliminary version of the questionnaire. Besides demographic and biological data, the resulting self- administered questionnaire included questions addressing knowledge (importance of colostrum, the average number of feeds the child should receive per day, up to what age the child should receive only breast milk and what age the mother should start supplementary food), questions addressing attitude (reasons for adopting breastfeeding, reasons for stopping breastfeeding, intention to breastfeed future children, intention to participate in classes related to breastfeeding in future pregnancy and the participant self-image) and questions addressing practice (time of commencement of breastfeeding after delivery, duration of breastfeeding, difficulties in initiating breastfeeding, age of starting formula, age at which breastfeeding was stopped and attending classes related to breastfeeding during pregnancy).

The responses were made as binary variables wherever applicable. The questionnaire was translated into Arabic. A panel of experts familiar with questionnaire development in the local Arabic dialect, comprising specialists in Clinical Medicine and in Evaluation and Measurement, was asked to assess the preliminary questionnaire and provide structured comments with respect to face and content validity, comprehensibility and comprehensiveness. The final version of the Arabic questionnaire was distributed, completed and collected from a pilot test group of female school teachers (not included in the final study). The data were analyzed and Cronbach’s alpha was used to assess internal consistency. Cronbach’s alpha was found to be 0.712, indicating an acceptable level of internal consistency of the final questionnaire.

## Ethical approval

The study received the approval of the Research and Ethics Committee of King Khalid University, College of Medicine (REC-2011-03-03).

### Data collection

Four hundred questionnaires were sent by regular mail to all the female schools within the Abha Female Educational District. Each school principal was requested to distribute the questionnaire to all the teachers who matched the criteria of having one child or more below the age of five years, with clear understanding of the voluntarily basis of their participation. By the end of the study period the school principal was requested to send back the completed questionnaires to the author. Their knowledge, attitude and practice of breastfeeding were assessed from their responses.

The inclusion criteria adopted was that each participant must have given birth to at least one child in the five years preceding the study. The participant responded to the questionnaire based on their experience with the last child.

### Analysis

Data were coded, validated and analyzed using *SPSS* PC + software package version 13. Descriptive statistical analyses were performed. Student *t* test and analysis of variance was used as test of significance at 5% level.

## Results

A total of 400 questionnaires were distributed, but only 384 participants returned completed copies, yielding a response rate of 96%. The mean age of the participants was 34.97 ± 4.32 years and their age ranged from 19 to 50 years.

### Description of the participants

The mean and standard deviation (SD) of the number of persons per household were 6.3 ± 1.9 persons, however, the number of children ranged from 1 to 6 children per family with mean of 2.6 **±** 0.9 child. The age range of the studied group was between 2 months and 5 years. The mean, standard deviation and median at which breastfeeding was stopped was found to be 8.7 ± 7.8 and 6 months respectively. Other selected characteristics of the participants are shown in Table 1.

**Table 1:** Selected characteristics of the participants

**Variable**	**Number**	**Percent (%)**
Age range of participants	19 to 50 y	
Mean age of participants	34.97 ± 4.32	
**Education background:**		
- University	222	57.8
- Teacher diploma	127	33.1
- Other	35	9.1
**Place of practice:**		
- Primary school	246	64.1
- Intermediate	89	23.2
- High school	49	12.8
Religion (Muslim)	384	100.0
**Husband education background:**		
- Primary	6	1.6
- Intermediate	31	8.1
- Secondary	124	32.3
- University	223	58.1
**Husband was employed:**		
- Yes	366	95.3
- No	18	4.7
Living with husband and children alone	353	91.9
Living with other member	31	8.1
Housemaid in the house	308	80.2
**Mode of delivery:**		
- Normal delivery	280	72.9
- Caesarean section	104	27.1
**Sex of the youngest child:**		
- Male	193	50.3
- Female	191	49.7
The age range of children related to this survey	2 months – 5 y	
The mean age of youngest child	2.52 ± 1.48 y	
The median age of youngest child	2 y	
**Importance of breast milk explained by physician:**		
- Yes	195	50.8
- No	189	49.2
The mean age and standard deviation at which breastfeeding was stopped	8.7 ± 7.8 months	
The median age when breastfeeding was stopped	6 months	

### Knowledge

Three hundred forty three (89.3%) of the participants reported that colostrum is good for the baby, while 5 (1.3%) consider it either not good or possibly detrimental to the child’s health and 36 (9.4%) don't know the answer. The duration of which the child should receive only breast milk without supplements was chosen by 108 (28%) to be 6 months, while 129 (33.5%) of participants think that the child must be breast fed exclusively up to 4–6 month of age (Table 2).

**Table 2:** Breastfeeding knowledge by female school teachers in Abha Female Educational District

**Variable**	**Number**	**Percent (%)**
**Knowledge**		
**Knowing the frequency of feeding on the first month:**		
- 8 times/day	74	19.3
- > 8 times/day	76	19.8
- Breastfeed on demand	207	53.9
- Not sure	27	7.0
**Knowing the advantage of colostrum:**		
- Good	343	89.3
- Not good	1	0.3
- May expose child to risk	4	1
- I don’t know	36	9.4
**Knowing the age when to introduce complementary food:**		
- 3 months	2	0.5
- 4 months	22	5.7
- 5 months	7	1.8
- 6 months	111	28.9
- 7 months	13	3.4
- 12 months	64	16.7
- 24 months	112	29.2
**Knowing the age up to which the child should receive only breast milk:**		
- 2 months	8	2.1
- 4 months	18	4.7
- 5 months	3	0.8
- 6 months	108	28.1
- 12 months	66	17.2
- 24 months	130	33.9

### Attitude

Plans to attend classes related to breastfeeding during pregnancy, driving force for starting breastfeeding and self image of the participants were the main questions addressing attitude (Table 3).

**Table 3:** Breastfeeding attitude by female school teachers in Abha Female Educational District

**Variable**	**Number**	**Percent (%)**
**Reasons behind adoption of breastfeeding:**		
- Religious background	225	58.6
- Child health	141	36.7
- Cleanliness and easy preparation	8	2.1
- Other reasons	10	2.6
**Intention to breastfed new child:**		
- yes	362	94.3
- no	22	5.7
**Plan to attend classes in future pregnancy:**		
- Intention to attend	261	68.0
- Not planning to attend	123	32.0
**Self image:**		
- Thin	11	2.9
- Normal	194	50.5
- Somewhat obese	153	39.8
- Obese	26	6.8

### Practices

The number of participants who breastfed between 4–6 months was 69 (17.9%).

With regards to giving formula, 349 of the respondents did give formula to their children as well as breast milk (90.9%), while 35 (9.1%) did not. The mean age and standard deviation of starting formula was 2.56 (± 2.5) months. In 256 participants (66.7%), the children were given readymade liquid formula while in hospital. The mean age at which breast milk was stopped was 8.7 (± 7.8) months with a median of 6 months (Table 4).

**Table 4:** Breastfeeding practice by female school teachers in Abha Female Educational District

**Variable**	**Number**	**Percent (%)**
**Practice**		
**Starting breastfeeding:**		
- Within one hour of delivery	119	31.0
- Within 6 hours of delivery	69	18.0
- After 6 hours but within 24 hrs of delivery	62	16.1
- After 24 hours of delivery but with 48 hours	134	34.8
Exclusive breastfeeding for 6 months	32	8.3
Breastfeeding between 4–6 months	69	17.9
**Baby given readymade liquid formula in hospital:**		
- Yes	256	66.7
- No	121	31.5
- Missing	7	1.8
Breastfeeding up to 2 y	15	3.9
No difficulty on initiation of breastfeeding	247	64.3
Giving both formula and breast milk	349	90.9
**Reasons for stopping breast milk (before 2 years)**		
- Breast milk was not sufficient	169	44.0
- Problem related to work place	148	38.5
- Child refusal	52	13.5
- Other reasons	15	3.9
**Classes attendance during pregnancy:**		
- Attended	33	8.6
- Not attended	351	91.4
**Follow-up during pregnancy:**		
- Regular	346	90.1
- Not regular	38	9.9

### Statistical analysis

The duration of exclusive breastfeeding was not significantly different between women who cited their religion as their motive for initiation of breastfeeding as compared to other motives (F = 0.212, p = 0.88). Similarly, the duration of exclusive breastfeeding was not significantly different between women who initiated breastfeeding in the first hour or later (F = 1.54, p = 0.202). The same pattern was also observed in the level of education of mothers and the period of exclusive breastfeeding (F = 0.912, p = 0.403).

## Discussion

The breastfeeding initiation rate, defined as the proportion of infants who received any breastfeeding whatsoever within the first 48 hours, was found to be 100%, which is similar to a study of health care workers in the same area (south western Saudi Arabia) [15]. Other Saudi Arabian studies have reported breastfeeding initiation rates ranging between 92 and 98% [9,11,12]. Only 119 of the participants (31%) complied with the WHO recommendations of starting breastfeeding within one hour of delivery [16].

Sustained exclusive breastfeeding up to six months without any supplement was reported by only 8.3% of participants. This figure is better than what has been reported by other Saudi Arabian [11] and US studies [17]. However, it is lower than the figure from a Ugandan study (49.8%) [18] and from a health care workers’ study (15.9%) [15].

The most important reason given by the participants for initiating breastfeeding was their Islamic religious background (56.6%), which is practiced by all of them. It is most likely related to the Islamic teaching in the Holy Quran which states “And mothers shall breastfeed their children for two whole years, for those who desire to complete the appropriate duration of breastfeeding” [7]. This finding is different from the health care workers’ study where the main reason was the child health (43.7%), followed by religious background (17.2%). It is worth mentioning that 55% of the health care workers in the study population were non-Muslim [15].

The breastfeeding was stopped at a mean age of 8.7 ± 7.8 months. The most common reason given for stopping breastfeeding was insufficiency of breast milk (44% of the participants), which is similar to other studies [12,19]. In fact, about 5% of women actually had physiologic insufficient milk supply, although up to (50%) or more reported that they perceived insufficient milk for their baby [20,21]. Thus, insufficient milk supply was considered to be more perceived than ‘real’ [22]. The next most important factor for early cessation of breastfeeding was work-related problems (38.5%). It is somewhat less than what was reported by the health care workers (45.7%) [15].

The effect of these two factors is likely to be the reason that most of the participants shifted to formula feeding by six month (91.7%). It also could explain, to some extent, the high rate of using readymade liquid formula while in hospital (66.7%). Such practice is to the contrary of WHO recommendations in the ten steps initiative for successful breastfeeding, and to the recommended practice of baby friendly hospitals [23,24]. This practice might be attributed to the willingness of the mother to train their baby to use formula from a young age due to the pressures of work environments unsuitable for breastfeeding.

It has been found that hospitals and work places without facilities for breastfeeding can undermine breastfeeding. Cohen and Mrtek showed that women employed by business establishments which were "breastfeeding friendly" were able to maintain a breastfeeding regimen for at least six months; rates comparable to those of women who were not employed outside the home [25].

Inadequate comprehensive maternity leave policies, lack of child care facilities at or near the workplace, rigid time schedules that do not allow for nursing breaks, lack of facilities providing privacy for breast-pumping and absence of facilities for refrigeration of pumped breast milk are among factors that affect breastfeeding prevalence among working mothers [26,27]. The maternity leave for working mothers in Saudi Arabia is very well outlined; she is entitled to 60 days leave with full salary and up to 3 years at 25 percent of her salary [28].

To breastfeed future children was the intention of the majority of participants (90.1%). It is similar to an earlier report from Saudi Arabia [10]. Such an attitude is one of the strongest predictors of breastfeeding initiation and duration [19].

Only a small number of participants (1.3%) disagreed with the idea of feeding colostrum or claimed ignorance of its benefits; similar to what was reported from other part of the country [29]. This finding was contrary to what was reported by Singh et al. in 1997, where 77% of mothers from the District of Rajasthan, India, discarded colostrum [30] and to what has been reported recently from other parts of India, where 15 to 60% of studied women still discard colostrum [31,32].

Low rates of knowledge regarding the appropriate duration of exclusive breastfeeding (28.9) and the time when complementary food should be introduced (30.7%), in conjunction with very low rates of attending classes related to breastfeeding issues during pregnancy (8.6%) are important factors in limiting breastfeeding prevalence. It also indicates the crucial role of health care providers and peer support to pregnant women and breastfeeding mothers. Such support, as well as face-to-face and pre- and postnatal classes, has been proven to be effective in reducing early cessation of breastfeeding and was a very effective way to promote breastfeeding prevalence [33,34].

Our study population, consisting of only female school teachers, may have limitations in not being applicable to other working Saudi women. Other factors which may limit generalization of our findings include cultural differences between this Educational District and other Saudi provinces and the inclusion of women whose youngest children were aged two months and five years, since this may affect accurate recall in responding to the questionnaire.

## Conclusion

This study revealed that breast milk insufficiency and adverse work related issues were the main reasons for the very low rate of exclusive breastfeeding among female school teachers in Abha educational district, Saudi Arabia. A very low rate of attending classes addressing the breastfeeding issues during pregnancy and an alarming finding of a high percentage of babies receiving readymade liquid formula while still in hospital were also brought out by the present study. Such findings, if addressed comprehensively by health care providers and decision-makers, will lead to improvement of child health in the study community.

## Competing interests

The author declares that he has no competing interests.
